# Impact of Salicylic Acid and PGPR on the Drought Tolerance and Phytoremediation Potential of *Helianthus annus*

**DOI:** 10.3389/fmicb.2018.02507

**Published:** 2018-10-23

**Authors:** Naeem Khan, Peiman Zandi, Shahid Ali, Asif Mehmood, Muhammad Adnan Shahid, Jianjun Yang

**Affiliations:** ^1^Department of Plant Sciences, Quaid-i-Azam University, Islamabad, Pakistan; ^2^Institute of Environment and Sustainable Development in Agriculture, Chinese Academy of Agricultural Sciences, Beijing, China; ^3^Plant Epigenetics and Development Lab, Northeast Forestry University, Harbin, China; ^4^Department of Botany, Abdul Wali Khan University Mardan, Mardan, Pakistan; ^5^Horticultural Sciences Department, Institute of Food and Agricultural Sciences, University of Florida, Gainesville, FL, United States

**Keywords:** PGPR, 16S RNA gene sequencing, salicylic acid, phytoremediation, heavy metals

## Abstract

The present study was aimed to isolate and characterize plant growth promoting rhizobacteria (PGPR) from the rhizosphere of rainfed area (Karak) in Pakistan. The influence of isolated rhizobacteria, in association with salicylic acid (SA), physiological attributes, drought tolerance potential, and phytoremediation in drought-stressed sunflower exposed was investigated. The isolated bacteria were named P1 and P2 and characterized on the basis of colony morphology and biochemical traits. Both PGPR P1 and P2 were identified on the basis of *16S-rRNA* gene sequencing as *Planomicrobium chinense* strain P1 (Accession No. MF616408) and *Bacillus cereus* strain P2 (Accession No. MF616406). The fresh cultures (24 h old) of isolates were used to soak the seeds pre-sowing. SA was foliar applied at three-leaf-stage. Likewise, the 30-days-old seedlings (three leaf stage) were exposed to drought stress. Drought stress was imposed to 30-days-old plants (three-leaf stage) by withholding water supply for the next 15 days until the soil water content reached 10%. The PGPR and/or SA treatment resulted in significant accumulation of Cd (84%), Pb (66%), and Ni (65%) in the rhizosphere. PGPR also induced accumulation of Cd and Ni in plant shoot. Combined treatment of PGPR and SA increased the Cu (21%), Co (11%), and Zn (8%) accumulation but decreased (12%) the Fe accumulation as compared to coinoculation of PGPR P1 and P2. Inoculation of plants with PGPR significantly increased shoot length (60%), root length (68%), root fresh (61%), and dry (63%) biomass under water stress. The inoculated plants had increased chlorophyll (67%), carotenoid (70%), leaf protein (64%), sugar (64%), and phenolic (62%) contents while lower leaf proline (62%) content, malondialdehyde (MDA) (64%), and antioxidant enzymes (67%) which suggest their role in drought tolerance. It is concluded that integrative use of PGPR in combination with SA found to be an efficacious strategy to improve the phytoremediation of heavy metals and plant growth under stressed conditions particularly under water-deficient conditions.

## Introduction

Various factors such as worldwide development, industrialization, agricultural practices, and anthropogenic activities are the most important factors responsible for soil pollution. These processes are responsible for the release of heavy metals causing serious ecological treat even if present in low concentration (Wuana and Okieimen, 2011). Recent technologies have been employed to eradicate the harmful metals from the rhizosphere which include leaching, soil excavation, fixation and landfill of the top contaminated soil but these techniques have high cost and secondary pollutants (Haque et al., 2008). Phytoremediation is an emerging *in situ* technology and more favorable due to its great potential. This includes the usage of plants to clean up the environments by reducing the toxicity, volume, and altering mobility of the contaminants in the soil (Shao-wei and Chang, 2004). Plants help to eliminate different pollutants such as pesticides, metals, oil, and other chemicals and play a central role in preventing the spread of contaminants through wind, rain or underground water from one area to another area. This technology comprised two important mutualistic components, i.e., plants and plant’s root microbes which degrade the toxic metabolites to non-toxic metabolites (Saxena et al., 2005).

Plant growth promoting rhizobacteria (PGPR) are the inhabitant microorganisms in the rhizosphere improving the success of phytoremediation. When the PGPR are inoculated into the contaminated soil, they enhance plant’s tolerance to heavy metals, accelerate the recycling of nutrients, modify toxic metals to less toxic, improve plant resistance against diseases and pest attack, and improve the soil structure (Khan and Bano, 2016; Karthik et al., 2017). The plant roots also secretes various exudates containing different free amino acids, carbohydrates, vitamins, and nutrients which are important for growth of rhizobacteria (Han et al., 2005; Babalola, 2010). PGPR serve many function for plant growth and development. They fix the atmospheric nitrogen, involved in the formation of several growth-promoting phytohormones. Moreover, the PGPR also contain enzymes that solubilize minerals which regulate plant growth (Glick et al., 1998; Ma et al., 2009; Oves et al., 2013a). In contrast to PGPR, salicylic acid (SA) is a synthetic plant growth regulator. It is economically feasible and cost effective, the SA induces systemic acquired resistance (SAR) in plants, hence protect the plants against pathogen attack and diseases, which in turn leads to improved plant growth.

Drought stress is a prevalent environmental restraint to plant growth and productivity. Drought is a meteorological term which means less water or rainfall (Khan et al., 2018). The drought stress resistance is seen in every plant but the extent is different in different plants (Jaleel et al., 2007). Water deficit and salt stresses are the global issues which effect the plant’s growth, water and nutrient relations, assimilate partitioning, photosynthesis, and respiration in plants. Reports indicated water deficiency effects plant’s at physiological, biochemical, and molecular characteristics such as cell division and expansion, leaf size, and fresh and dry weights (Ali et al., 2014; Glick, 2014). The key mechanisms by which PGPR encourage plant growth under stress condition includes surge in the level of endogenous ethylene concentration through 1-aminocyclopropane-1-carboxylate (ACC) deaminase enzyme, improve photosynthetic pigments, invigorating root growth, rhizoremediation, and disease resistance (Belimov et al., 2005; Madhaiyan et al., 2006).

Sunflower (*Helianthus annuus* L.) is an annual crop and belongs to family *Asteraceae*. This is a quickly growing plant and used as a target species with great potential to accumulate the heavy metals in roots, stem, and leaves. It is demonstrated that sunflower accumulates many heavy metals (Zn, Cu, and Pb) in shoot. However, some reports indicates that sunflower accumulate heavy metals in roots and restrict their translocation to above ground parts (Madejon et al., 2003). It has been found that metals translocate very effectively from root to upper plant parts (Marchiol et al., 2007). It was hypothesized that PGPR and SA can improve the phytoremediation and drought tolerance potential in sunflower under water-deficient conditions. Therefore, this investigation was executed to isolate drought tolerant PGPR strains from rainfed area of Karak and then evaluate their individual role as well as in combination with SA, on the growth and phytoremediation potentials in drought-stressed sunflower. PGPR and SA enhance the process of phytoremediation and drought tolerance in plants.

## Materials and Methods

The experimental work was carried out in the green house at the Department of Plant Sciences, Quaid-i-Azam University Islamabad, Pakistan (33.7294° N, 73.0931° E, average temperature of 24°C, and humidity of 53%) in sunflower growing season 2015–16. Seeds of sunflower (Pioneer 6480) were sown in plastic pots (30 cm × 40 cm) and filled with soil and sand (1500 g) in a ratio of 3:1. The soil used in pots was collected from the agricultural field (sunflower) at Karak that has a pH of 6.8. The soil contained total dissolved solids (TDS) = 74 mg/50 ml, organic matter = 17.1 g/kg, N = 1.73 g/kg, P = 0.71 g/kg, K = 19.3 g/kg, Pb = 313.7 mg/kg, Cd = 5.7 mg/kg, Ni = 60 mg/kg, Zn = 1.14 mg/kg, Cu = 1.01 mg/kg, Co = 0.88 mg/kg, and soil conductivity was 3.68 μS/cm. Seeds of sunflower were washed with distilled water and surface sterilized with 95% ethanol for 2 min and then treated with 10% clorox before sowing. The SA was applied once at three-leaf stage (25 ml/pot). The pots were kept well-watered throughout the experiment until the start of drought stress. Pots were watered until the water seep out from the bottom of pots. Drought stress was applied at three leaf stage by withholding the supply of water for 15 days until the soil water content reached to 10%. Colonies of bacterial isolates were secluded from the rhizosphere of sunflower grown in the rainfed area at Karak, Pakistan. The selected colonies were named as P1 (*Planomicrobium chinense* strain P1) and P2 (*Bacillus cereus* strain P2) and evaluated for their plant growth promoting and phytoremediation effect under water-deficient condition. The experiment was arranged as complete randomized design (CRD) with four replications. The experiment had nine treatments which are described below (Table 1):

**Table 1:** Experimental work plan.

Symbol	Treatments	Symbol	Treatments
T1	Seeds treated with PGPR P1	T2	Seeds treated with PGPR P1 + sprayed with SA
T3	Seeds treated with PGPR P2	T4	Seeds treated with PGPR P2 + sprayed with SA
T5	Seeds treated with PGPR P1 and P2	T6	Seeds treated with PGPR P1, P2 + sprayed with SA (consortium)
T7	Plants treated with SA (foliar spray)	T8	Irrigated C (uninoculated and irrigated plants)
T9	Stress C (uninoculated untreated plants grown under stress)		

*Where P1: Planomicrobium chinense strain P1; P2; Bacillus cereus strain P2.*

### Isolation and Inoculation Procedure of PGPR

Soil samples were collected from the rhizosphere of 3-months-old sunflower plants at the depth of about 12 cm by uprooting the plants. The soils from the roots were collected by shaking in clean sterilized ziplock bags. The collected soil samples were immediately transported to the laboratory for decimal dilution and aliquots were transferred to the Luria Bertani (LB) media from the rhizosphere soil on which sunflower was growing. Morphologically dissimilar colonies were carefully chosen and purified with further streaking. The culture thus obtained was incubated in a shaker, centrifuged, and the obtained pellet was deferred in distilled water and the optical density (at 660 nm) was adjusted to be 1, which was equal to 10^6^ cells/ml. This suspension was used for seed inoculation prior to sowing.

### Characterization of Bacterial Isolates

#### Colony and Cell Morphology

The isolates were incubated on agar plates and the final cultures (24 h old) were utilized for identification of isolated bacterial strains under microscope (Bio-Microscope XSZ-701, China). The color and shape of the colonies were recorded (Miller and Schroth, 1972).

#### Catalase and Oxidase Test

The catalase (CAT) and oxidase tests were performed by using the method of Steel (1961) and MacFaddin (1980). Briefly, 24-h-old bacterial cultures were dropped on the slide following the addition of 30% hydrogen peroxide (one drop). CATs were recorded based on the existence of gas bubbles (MacFaddin, 1980). To test the presence of oxidase in bacterial cell, Kovac’s reagent (Kovacs, 1956) was made by the addition of hot distilled water. A strip of filter paper was immersed in this reagent and then desiccated. Twenty-four-hours-old bacterial colonies were transferred to filter paper with a color change from purple to black indicating positive peroxidase activity.

#### Phosphorous Solubilization Index (PSI)

The phosphorous solubilization index (PSI) was calculated as described by Pikovskaya (1948). A sterilized Petri-plate was filled with Pikovskaya’s media and allowed to solidify for 30 min. A pin point inoculum was transferred to solidified Pikovskaya media. The Pikovskaya media with inoculum was incubated at 28°C for 7 days. After that, the diameter of the colony was measured and the solubilization index was calculated as:

SI=diameter (cm)+halozone (cm)=diameter (cm)

#### Antibacterial and Antifungal Activities

An agar well-diffusion method was used for the determination of antibacterial activity (Navarro et al., 1996). The bacterial strains used for this study were included *Staphylococcus aureus*, *Pseudomonas aeruginosa*, *Klebsiella pneumoniae*, and *Escherichia coli*. Whereas for antifungal activity, we followed agar tube dilution method as described by Washington and Sutter (1980). The two fungal strains, i.e., *Helminthosporium sativum* and *Fusarium solani* were used for this study.

#### Heavy Metal Tolerance

The selected bacterial strains were tested for their resistance to heavy metals by agar dilution method (Cervantes-Vega et al., 1986). Freshly prepared agar plates were amended with various soluble heavy metal salts namely Cd, Pb, and Ni at various concentrations ranging from 30 to 1500 mg/l were inoculated with overnight grown cultures. Heavy metal tolerance was determined by the appearance of bacterial growth after incubating the plates at room temperature for 48 h.

#### Viable Cell Count Method (cfu)

For determination of colony forming unit (cfu), decimal dilutions from the collected soil sample was made and viable cell counts were calculated as suggested by Khan and Bano (2016):

Viable cell count (CFU/g)=(no. of colonies×dilution factor/volume of inoculum)

(No. of colonies × dilution factor/volume of inoculum).

### Extraction of Bacterial DNA and *16S rRNA* Sequence Analysis

For extraction of bacterial DNA, tryptone yeast (TY) extract broth was inoculated with a single bacterial colony. The inoculated TY broth was incubated overnight in shaker (Model: Excella E-24). The instant grown culture was centrifuged at 12,000 rpm for 10 min followed by suspending in lysis buffer. This was followed by the addition of 5 M NaCl (60 ml) and again centrifuged for 10 min at 12,000 rpm. The supernatant was shifted into a new tube trailed by the addition of chloroform. The centrifugation was done two times after adding 100% ethanol to clean the attained DNA. The obtained DNA was assessed through nanodrop spectrophotometry (260–280 nm; Chen and Kuo, 1993). The cleaned DNA was than amplified by polymerase chain reaction (PCR) following the procedure of Weisburg et al. (1991). The 1 kb DNA ladder (Fermentas, Germany) was used as molecular marker. The cleaned fragments of 1400 bp were sequenced and the sequenced products were determined on an Applied BioSystems model 3730XL automated DNA sequencing system at the Macrogen, Inc., Seoul, Korea.

### Physiological and Biochemical Analyses of Plants

#### Chlorophyll and Leaf Proline Content

The chlorophyll content in the leaves of sunflower was estimated by chlorophyll meter (Spad-502 plus, Serial No. 20001472 made by Konica Minolta, Japan). The Proline content of sunflower leaves was determined following the method of Bates et al. (1973).

#### Malondialdehyde (MDA) Content

The lipid peroxidation estimated as malondialdehyde (MDA) content was recorded by calculating the amount of MDA formed by thiobarbituric acid (TBA) reaction as defined by Cui et al. (2000).

#### Leaf Protein Content

Leaf protein content was estimated based on the method of Lowry et al. (1951). The leaf samples were ground in 1 ml phosphate buffer and centrifuged at 3000 rpm. The supernatant was transferred to new tubes and total volume was made to 1 ml by the addition of distilled water. The solution was mixed by shaking after adding reagent C and D and incubated for half an hour at room temperature. The absorbance of each sample was determined at 650 nm against different concentrations of bovine serum albumen (BSA). Protein concentration was calculated as:

Protein concentration mg/g=K value×dilution factor×absorbance/sample wt

K value = 19.6, dilution factor = 2, wt. of sample = 0.1 g

#### Sugar Estimation

Sugar contents were determined by the method of Dubois et al. (1956). Leaf tissues were ground with distilled water (10 ml) then centrifuged at 3000 rpm for 5 min. The supernatant (0.1 mL) was mixed with 1 ml phenol (80%) and 5 ml concentrated H_2_SO_4_. The absorbance was recorded at 420 nm. The concentration of the unidentified sample was considered with reference to the standard curve made by using glucose:

Sugar concentration mg/g=K value×dilution factor×absorbance/sample wt

K value = 20, dilution factor = 10, wt. of sample = 0.5 g

#### Total Phenolic Content

Folin-Ciocalteu colorimetric method (Barka et al., 2006) was used for determination of total phenolic content. Fresh leaves (600 mg) were ground with 80% ethanol (5 ml) by using homogenizer. The grounded samples were poured into 50 ml tightly covered plastic tubes and filtered after incubating for 2 h at 4°C in the dark. Ethanol (2.5 ml) was added to the pellet. Four replicates consisting of leaf extract (125 μl), Folin-Ciocalteu reagent (625 μl), and 7.5% (wt/vol) Na_2_CO_3_ (250 μl) were vortexed for few seconds and incubated (45°C) for 15 min in a water bath. Phenolics were measured at 750 nm using gallic acid as the standard and were expressed as mM GA eq/g FW.

#### Extraction of Antioxidant Enzymes

A 0.5 g of fresh leaf tissue was used for the determination of antioxidant enzymes. The fresh leaf tissue was ground in a phosphate buffer (5 mL of 50 mM) in an ice bath. Then the mixture was centrifuged for 20 min at 13,000 rpm. The supernatant was collected and utilized for different enzyme assays.

For peroxidase (POD, EC# 1.11.1.x) determination, the modified method of Gorin and Heidema (1976) was used. For the determination of POD activities, an assay mixture was prepared by adding 1.35 μL MES buffer (pH 5.5), 0.05% H_2_O_2_, and 0.1% phenylene diamine (1 μL) with an enzyme extract of 0.1 mL. Absorbance at 485 nm was recorded in a spectrophotometer. Change in optical density was recorded at 485 nm/min as one unit of POD.

We followed the procedure of Asada and Takahashi (1987) to determine ascorbate peroxidase (APOX) activity (EC# 1.11.1.11). A reaction mixture was prepared by adding 50 mM KH_2_PO_4_ buffer, 0.5 mM ascorbic acid, and H_2_O_2_ (0.1 mM) to a 100 μL enzyme extract. A blank solution was made the same way but without the enzyme extract and absorbance was recorded at a wavelength of 290 nm.

Catalase (EC# 1.11.1.6) activity was estimated following the method of Chandlee and Scandalios (1984), where an assay mixture was prepared by adding KH2PO4 (1 mM) buffer (2.6 mL) and H_2_O_2_ (400 μL) with an enzyme extract (40 μL). The breakdown of H_2_O_2_ was an indicator of the CAT activity in the leaves and was recorded by the absorbance of light by H_2_O_2_ at 240 nm, and the CAT activity was expressed in l U/mg protein (U = 1 mM of H_2_O_2_ reduction/min/mg of protein).

#### Determination of IAA, GA3, and ABA

Phytohormone were extracted and purified from plant samples following the method described by Khan and Bano (2016). Methane (80%) along with butylated hydroxyl toluene was used for crushing the fresh leaves (1 g). The extract was centrifuged (3000 rpm) and supernatant was dispensed with ethyl acetate. Rotary film evaporator (REF) was used to dry the ethyl acetate phase and the residues were resuspended in methanol. The obtained samples were analyzed on HPLC after filtering through Millipore filter. IAA and GA3 were extracted at 280 and 254 nm wavelength separately, whereas for ABA, the samples were inserted into C18 column and eluted with a linear gradient of methanol (30–70%), having 0.01% acetic acid, at a flow rate of 0.8 ml/min.

#### Shoot and Root Length (cm)

Shoot and root length was recorded for five randomly selected plants per treatment with the help of meter rod.

#### Root Fresh and Dry Weight (g)

The root samples were first dried at 60°C for 48 h and then both the fresh and dried root samples were weighed with the help of an electronic balance.

### Nutrients Analysis of Rhizosphere Soil

Ammonium bicarbonate-diethylene triamine penta acetic acid (DTPA) method was used for the nutrient analysis (micronutrients Cu, Co, Fe, and Zn and heavy metals Cd, Pb, and Ni) of rhizosphere soil developed by Soltanpour and Schwab (1977).

### Nutrient Analysis of Plant Leaves

Dried leaves (0.25 g) of plant sample digested in a 50 ml flask with a solution of nitric acid, sulfuric acid and perchloric acid in a ratio of 5:1:0.1, respectively. The mixture was boiled on hot plate under a fume hood until digestion was completed, which was indicated by the presence of white fumes from the flasks. The sample was then allowed to cool by adding distilled water till a final volume of 50 ml. Whatman No. 42 filter paper was used for filtering the extract and were analyzed by atomic absorption spectrophotometer (Shimadzu AA-670) for the presence of various metals (Allen et al., 1974).

### Data Analysis

The data were analyzed using SAS version. 9.1. An ANOVA was performed to determine the effect of treatments and error associated with the experiment with replications and treatments as random effects. To identify significant differences among treatments, a mean comparison was carried out by using protected LSD (*P = 0.05*) test where error mean square was used to estimate the standard error of differences between mean.

## Results

### Colony Forming Unit (cfu) of Rhizosphere Soil

The rhizosphere of plants inoculated with *B. cereus* strain P2 exhibited greater cfu than that of *P. chinense* strain P1. Addition of SA increased the cfu significantly for both the PGPR. Coinoculation of P1 and P2 was more effective than their individual application. Significant increase in cfu was noted in plants from inoculated seeds and sprayed with SA followed by those having seed inoculation only (i.e., T6 followed by T5; Table 2).

**Table 2:** Colony forming unit of PGPR in the rhizosphere.

Symbol	Treatments	CFU g^−1^ of soil
T1	Seeds inoculated with P1	9 × 10^5^
T2	Seeds inoculated with P1 and sprayed with SA	9.9 × 10^5^
T3	Seeds inoculated with P2	10.4 × 10^5^
T4	Seeds inoculated with P2 and sprayed with SA	11.1 × 10^5^
T5	Seeds inoculated with P1 + P2	14.6 × 10^5^
T6	Seeds inoculated with P1 + P2 and sprayed with SA	14.9 × 10^5^
T7	Foliar spray of SA	0 × 10^5^
T8	Irrigated C	0 × 10^5^
T9	Stress C	0 × 10^5^

*T1, seeds treated with PGPR P1; T2, seeds treated with PGPR P1 and sprayed with SA; T3, seeds treated with PGPR P2; T4, seeds treated with PGPR P2 and sprayed with SA; T5, coinocualtion of PGPR P1 and P2; T6, seeds treated with PGPR P1 and P2 + sprayed with SA; T7, foliar application of SA; T8, untreated uninoculated irrigated C; T9, untreated uninoculated stress C (P1: Planomicrobium chinense strain P1; P2; Bacillus cereus strain P2).*

### Antibacterial and Antifungal Activities of PGPR Isolates

*Planomicrobium chinense* strain P1 and *B. cereus* strain P2 had significant activity against all four used bacterial strains, i.e., *S. aureus*, *P. avanigadda*, *K. pneumoniae*, and *E. coli*. The *B. cereus* strain P2 had no response against *E. coli*. Maximum mycelial inhibition (65%) of *H. sativum* was observed under *B. cereus* strain P2. Whereas *P. chinense* strain P1 was less effective against *H. sativum*, but significantly (78%) inhibited growth of *F. solani* (Table 3).

**Table 3:** Antibacterial and antifungal activities of selected PGPR strains.

	Antibacterial activities					Antifungal activities	
S. No.	Isolates	*S. aureus*	*P. aeruginosa*	*K. pneumoniae*	*E. coli*	*H. sativum*	*F. solani*
1	*P. chinense* strain P1	+	+	+	+	36% b	78% a
2	*B. cereus* strain P2	+	+	+	−	65% a	69% b

*Different letter (i.e., a and b) indicate significant differences (P < 0.05) among treatments.*

### Phosphate Solubilization Index

*Planomicrobium chinense* strain P1 and *B. cereus* strain P2 proved P-solubilizer but *B. cereus* strain P2 presented the efficient phosphorus solubilizing potential with phosphorus solubilization index of 2.99. However, phosphorus solubilization index for *P. chinense* strain P1 was 1.13 (Table 4).

**Table 4:** P-solubilizing activity of selected PGPR strains.

S. No.	Isolates	Halozone dm (mm)	P-solubilization index (mm)
1	*P. chinense* strain P1	0.29 a	1.13 b
2	*B. cereus* strain P2	0.95 a	2.99 a

*Different letter (i.e., a and b) indicate significant differences (P < 0.05) among treatments.*

### Antibiotic Resistance of Selected PGPR Strains

*Bacillus cereus* strain P2 presented higher tolerance (200 μg/ml) to chloramphenicol than *P. chinense* strain P1 (100 μl/ml). Whereas both isolated PGPR species were able to tolerate streptomycin (200 μg/ml). The *P. chinense* strain P1 *had* tolerance to hygromycin B up to 150 μg/ml but *B. cereus* strain P2 did not tolerate beyond 100 μg/ml (Table 5).

**Table 5:** Antibiotic resistance of selected PGPR strains.

S. No.	PGPR	Chloramphenicol (μg/ml)						Streptomycin (μg/ml)						Hygromycin B (μg/ml)					
		25	50	75	100	150	200	25	50	75	100	150	200	25	50	75	100	150	200
1	*P. chinense* strain P1	+	+	+	+	−	−	+	+	+	+	+	+	+	+	+	+	+	−
2	*B. cereus* strain P2	+	+	+	+	+	+	+	+	+	+	+	+	+	+	+	+	−	−

### Heavy Metal Tolerance

The selected PGPR strains were checked for their tolerance ability against Cd, Pb, and Ni. Both the PGPR strains were found effective against the heavy metals and showed maximum tolerance. *P. chinense* strain P1 showed maximum tolerance (i.e., 30–1500 mg/l) to heavy metals except Ni where it was ineffective beyond 1500 mg/l, whereas *B. cereus* strain P2 was ineffective against Pb and Ni at concentration of 1500 mg/l (Table 6).

**Table 6:** Heavy metal tolerance ability of selected PGPR strains.

S. No.	PGPR	Heavy metal tolerance (mg/l)																	
		Cd						Pb						Ni					
		30	100	200	500	1000	1500	30	100	200	500	1000	1500	30	100	200	500	1000	1500
1	*P. chinense* strain P1	+	+	+	+	+	+	+	+	+	+	+	+	+	+	+	+	+	−
2	*B. cereus* strain P2	+	+	+	+	+	+	+	+	+	+	+	−	+	+	+	+	+	−

### Heavy Metal Accumulation in Rhizosphere and Plant Shoot

**FIGURE 1:**
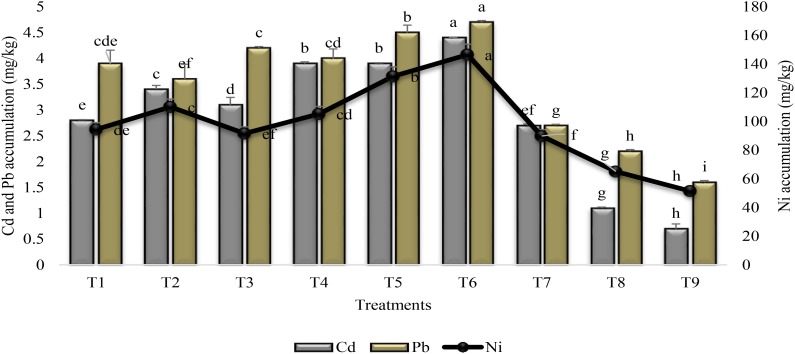
Heavy metal accumulation in the rhizosphere of sunflower grown under drought and control conditions and treated with PGPR and SA. Error bars represent standard errors of the mean (*n* = 4) at each time point. Different letters indicate significant differences (*P* < 0.05) among treatments. T1, seeds treated with PGPR P1; T2, seeds treated with PGPR P1 and sprayed with SA; T3, seeds treated with PGPR P2; T4, seeds treated with PGPR P2 and sprayed with SA; T5, coinocualtion of PGPR P1 and P2; T6, seeds treated with PGPR P1 and P2 + sprayed with SA; T7, foliar application of SA; T8, untreated uninoculated irrigated C; T9, untreated uninoculated stress C (P1: *Planomicrobium chinense* strain P1; P2: *Bacillus cereus* strain P2).

There was substantial effect of treatments on the accumulation of heavy metals in the rhizosphere (Figure 1). Combined application of PGPR and SA (T6) significantly enhanced the heavy metal accumulation than stress control (T9). Both the PGPR and SA treatments enhanced Cd accumulation (84%) in the rhizosphere but the increase was 66 and 65% for Pb and Ni as compared to stress control. The treatmental set, seeds inoculated with PGPR P2 coupled with SA (T4), and coinoculation of seeds with PGPR P1 and P2 (T5) were at par for the Cd accumulation in the rhizosphere. Seed inoculation with PGPR P1 promoted the accumulation of Ni, whereas higher accumulation of Pb and Cd was recorded in the rhizosphere of plants treated with P2. Application of SA reduced the PGPR P1 and P2 induced increase in heavy metal accumulation (i.e., T1 and T3 vs T2 and T4). SA alone (T7) was found to less effective in increasing heavy metal accumulation in the rhizosphere in comparison to inoculation with PGPR.

**FIGURE 2:**
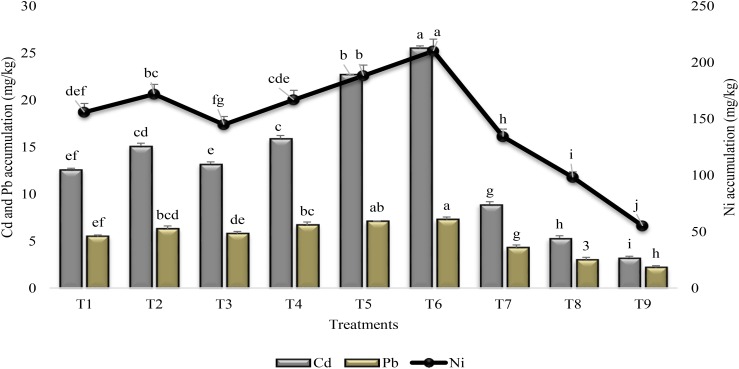
Heavy metal accumulation in the shoot of sunflower grown under drought and control conditions and treated with PGPR and SA. Error bars represent standard errors of the mean (*n* = 4) at each time point. Different letters indicate significant differences (*P* < 0.05) among treatments.

Foliar application of SA and PGPR significantly enhanced the Cd and Ni accumulation in the shoot of all inoculated plants over untreated uninoculated plants grown under stress condition (T9) and over irrigated control (T8) (Figure 2). Maximum increase (87 and 74%) in Cd and Ni accumulation was recorded when PGPR and SA were mutually applied (T6) followed by the collective inoculation with P1 and P2 (T5). The collective application of PGPR and SA augmented the Pb accumulation with 60% more in shoots than those of control plants. Inoculation with P1, in combination with SA, (T2) was more effective (17%) for Cd accumulation than individual inoculation with P1 (T1). Mixed inoculation of seeds with P1 and P2 was more effective for accumulating Cd and Ni than individually application with P1 or P2. All the treatments found to be ineffective in case of accumulation of Pb. Foliar application of SA (T7) was more effective for accumulating Ni than Cd and Pb by enhancing the Ni accumulation (59%) in shoot of plants. In general, heavy metal accumulation was significantly enhanced in combined treatment of two PGPR or PGPR in combination with SA.

### Micronutrient Accumulation in Rhizosphere and Plant Shoot

**FIGURE 3:**
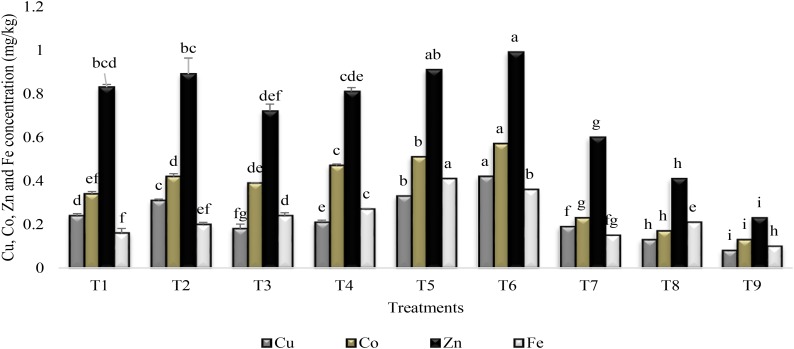
Micronutrients accumulation in the rhizosphere of sunflower grown under drought stress and control conditions and treated with PGPR/SA. Error bars represent standard errors of the mean (*n* = 4) at each time point. Different letters indicate significant differences (*P* < 0.05) among treatments.

All the treatments significantly enhanced the accumulation of Cu, Co, Fe, and Zn over the control (T9) in the rhizosphere of sunflower (Figure 3). The coinoculation of PGPR P1 and P2 (T5) significantly enhanced (75%) the accumulation of Fe as compared to stress control. Combined application of PGPR and SA (T6) was more effective for increasing the Cu (81%), Co (77%), and Zn (77%) accumulation. Plants receiving the treatment of SA and inoculated with PGPR P2 enhanced Fe and Cu accumulation in the rhizosphere by 76% as compared to stress control. They also had significantly enhanced (75%) Zn and Co accumulation over control. Inoculation with PGPR P1, applied alone (T1) or with SA (T2) increased Zn accumulation (72%) over stress control but was less effective for Fe. SA alone was more effective for increase in Zn (61%) and Cu (58%) accumulation but was less effective for Fe and Co accumulation in rhizosphere.

**FIGURE 4:**
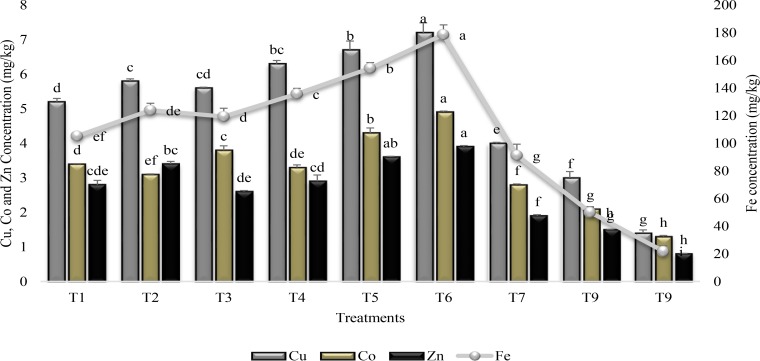
Micronutrients accumulation in the shoot of sunflower grown under drought and control conditions and treated with PGPR and SA. Error bars represent standard errors of the mean (*n* = 4) at each time point. Different letters indicate significant differences (*P* < 0.05) among treatments.

Similarly, both the SA and PGPR either used alone or in combination increased the micronutrient accumulation in shoot of plants over stress control (T9) and irrigated control (T8) (Figure 4). Combine application of PGPR and SA (T6) was the most efficacious for micronutrient accumulation in plant shoot. PGPR in combination with SA (T6) significantly increased Fe accumulation (88%) in plant shoot whereas the increase was 81, 79, and 73% for Cu, Zn, and Co, respectively, in comparison to control (T9). Inoculation with PGPR P1 and P2 alone or in combination significantly enhanced (>78%) the Fe accumulation over T9. However, inoculation with PGPR P2 was more effective than PGPR P1 for Cu and Co accumulation, whereas P1 accumulated more Zn than P2. SA alone was effective for accumulating more Fe (76%) and Cu (65%) than control (T9).

### Chlorophyll and Carotenoids Content

Chlorophyll and carotenoids content were significantly decreased by 64 and 47%, respectively, in leaves of drought stressed plants than those under control (Table 7). However, plants treated with PGPR and/or SA showed lower reductions compared to untreated plants grown under drought stress. Treatments T5 (coinoculation of PGPR P1 and P2) and T6 (consortium of PGPR/SA) had significantly increased the leaf chlorophyll content even greater than control. SA alone was also effective in improving the chlorophyll content and was at par with T1 (plants treated with PGPR P1). The carotenoids content showed variable results and in all the treated plant relative to stressed and irrigated controls.

**Table 7:** Values of leaf chlorophyll and carotenoids contents, proline, MDA, protein and phenolics content, and antioxidant enzyme activities in the leaves of sunflower.

Treatments	Chlorophyll	Carotenoids	Proline	Protein	MDA (nmol/g fwt.)	Sugar (mg/g)	Phenolics (mg GAE/g)	Catalase	APOX	SOD
					
	(μg/g)							(units/g fwt.)		
T1	33.2	4.3	0.21	1.6	0.16	2.9	2.8	0.27	0.15	0.39
T2	36.4	4.8	0.17	1.9	0.13	3.1	3.2	0.21	0.13	0.34
T3	31.3	4.6	0.24	1.4	0.14	2.4	3.1	0.31	0.18	0.43
T4	35.7	5.1	0.2	1.8	0.12	2.7	3.7	0.25	0.15	0.37
T5	39.3	5	0.16	2.1	0.12	3.4	3.9	0.21	0.12	0.27
T6	42.1	5.6	0.14	2.5	0.1	3.9	4.3	0.14	0.1	0.23
T7	33.2	3.9	0.26	1.5	0.15	2.3	2.7	0.25	0.17	0.42
T8	38.4	3.2	0.13	2.3	0.09	3.5	4.3	0.11	0.07	0.19
T9	13.9	1.7	0.37	0.9	0.28	1.4	1.6	0.49	0.35	0.58

*MDA, malondialdehyde; APOX, ascorbate peroxidase; SOD, superoxide dismutase; T1, seeds treated with PGPR P1; T2, seeds treated with PGPR P1 and sprayed with SA; T3, seeds treated with PGPR P2; T4, seeds treated with PGPR P2 and sprayed with SA; T5, coinocualtion of PGPR P1 and P2; T6, seeds treated with PGPR P1 and P2 + sprayed with SA; T7, foliar application of SA; T8, untreated uninoculated irrigated C; T9, untreated uninoculated stress C (P1: Planomicrobium chinense strain P1; P2; Bacillus cereus strain P2).*

### Leaf Proline and MDA Contents

The leaf proline and MDA contents were significantly enhanced in untreated plants grown under stress condition (Table 7). However, PGPR and SA treatment (T6) significantly reduced the leaf proline and MDA contents. The maximum decrease by 65 and 64% in leaf proline and MDA contents, respectively, was noted in T6 (consortium of PGPR and SA) followed by T5 (coinoculation of PGPR P1 and P2). PGPR P1 and P2 in association with SA (i.e., T2 and T4) were more effective in reducing the proline and MDA contents than P1 and P2 used alone. Treatment T4 (PGPR P2 in association with SA) was at par with T5 for MDA content. SA alone (T7) was also effective in reducing (30 and 46%) the leaf proline and MDA contents; however, reduction was lower than PGPR inoculation.

### Leaf Protein, Sugar, and Phenolics Content

Drought stress caused significant changes in leaf protein, sugar, and phenolics content in sunflower leaves (Table 7). The leaf protein content was decreased by 61% under drought stress compared to irrigated control, whereas the leaf sugar and phenolics content was decreased by 60 and 63%, respectively. The combined application of PGPR and SA (T6) considerably enhanced the leaf protein, sugar, and phenolics content and the increase was even greater (8 and 10%) than irrigated control for protein and sugar contents, whereas leaf phenolics content of T6 was at par with irrigated control. PGPR P1 was more effective for leaf protein and sugar content than P2, whereas P2 was more responsive for enhancing the leaf phenolics content. SA alone or in association with PGPR was effective in increasing the leaf protein, sugar and phenolics contents and significantly enhanced (40, 39, and 41%) the leaf protein, sugar, and phenolics content, respectively.

### Antioxidant Enzymes

Drought stresses caused significant increase in the activities of antioxidant enzymes but were minimal in the irrigated control (Table 7). Combined treatment of PGPR and SA significantly reduced the activities of antioxidants when applied alone or in combination. Combined treatment of PGPR and SA (T6) was more effective in reducing the antioxidant enzymes as compared to PGPR or SA alone. Maximum decrease (71%) was noted in the activities of CAT and APOX, whereas the SOD activity was reduced by 60% as compared to untreated drought stress plants. Combined treatment of PGPR P1 and P2 (T5) was more effective than P1 and P2 alone and significantly reduced (65, 57, and 53%) the APOX, CAT, and SOD activities. Treatment T2 was at par with T5 and T4 = T7 for CAT activity, whereas T1 = T4 for APOX activity. The SA alone was also effective in reducing the CAT (49%) and APOX (51%) activities but was less effective in reducing the SOD activity.

### Growth Parameters

**FIGURE 5:**
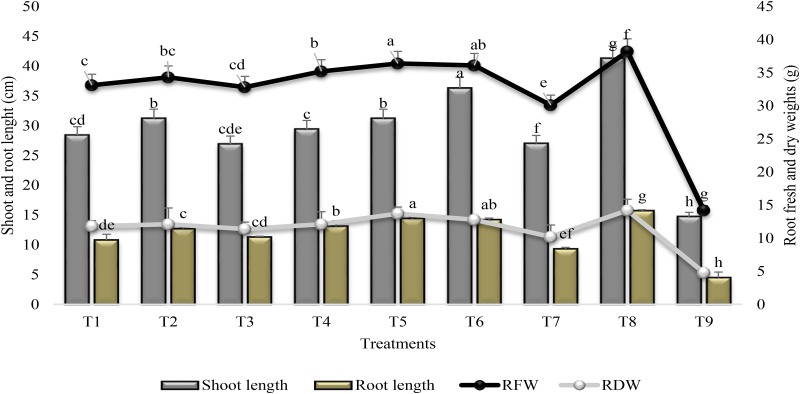
Shoot and Root length and root fresh and dry weights (±SE) of sunflower grown under drought and control conditions and treated with PGPR and SA. Error bars represent standard errors of the mean (*n* = 4) at each time point. Different letters indicate significant differences (*P* < 0.05) among treatments.

Plant growth promoting rhizobacteria treatment significantly increased shoot and root length and plant biomass (Figure 5). SA alone (T7) had no significant effect on root length and root dry weight as compared to PGPR inoculated plants. Maximum increase (60 and 67%) in shoot and root length was recorded in T6 (combined treatment of PGPR and SA) as compared to stress control (T9) though the increase was less (12 and 10%) than irrigated control. Combined treatment of PGPR P1 and P2 was more effective for increase (61 and 65%) in root fresh and dry weights followed by T6. Drought stress reduced (63 and 66%) the root fresh and dry weights significantly in the test plant; however, combined treatment of PGPR P1 and P2 significantly reduced (≤5%) the damage caused by drought stress. Treatment T2 was at par with T5 for shoot length and, T2 = T4 for root dry weight. Application of SA stimulated the shoot length (46%) and root fresh weight (53%) as compared to stress control.

### IAA, GA, and ABA Content

**FIGURE 6:**
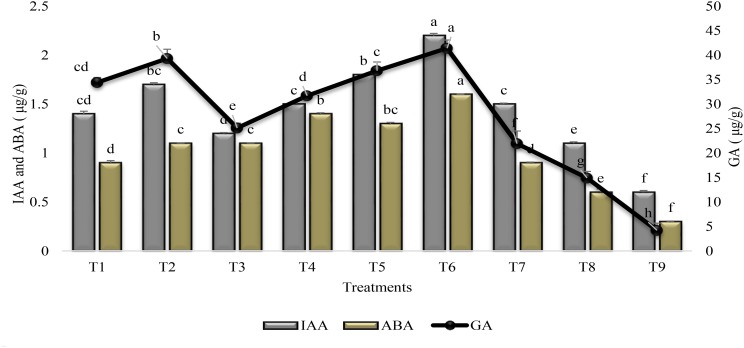
Phytohormone content (±SE) in leaves of sunflower grown under drought and control conditions and treated with PGPR and SA. Error bars represent standard errors of the mean (*n* = 4) at each time point. Different letters indicate significant differences (*P* < 0.05) among treatments.

Phytohormones were significantly improved in response to all treatments as compared to stress and irrigated control plants (Figure 6). The mutual application of PGPR and SA (T6) increased (>70%) the IAA, GA, and ABA content in the leaves of sunflower as compared to untreated uninoculated plants grown under drought stress condition (T9). The % increase was higher (89%) for GA as compared to IAA (73%). PGPR P1 alone (T1) or in association with SA (T2) was more effective in increasing the phytohormone content than P2 (T3 and T4). Combined treatment of SA along with PGPR P1 (T2) was more effective for GA than coinoculation of P1 + P2 (T5). Foliar application of SA (T7) was more effective for increasing (81%) the GA content, whereas the increase was 66 and 60% for ABA and IAA contents. Combined treatment of SA and PGPR P1 or P2 was at par with P1 alone and was less effective than P2 for ABA content but was more effective for IAA and GA contents.

## Discussion

Various anthropogenic activities are responsible for heavy metals accumulation in the environment and consequently contaminate the food chain (Ali et al., 2013). So, there is a dire need to remediate these metals by the use of various hyper-accumulators. Hyper-accumulators are capable to grow in soils having high metal concentration and have the ability to remediate the heavy metals from contaminated soils (Pence et al., 2000). Many plant species coupled with exogenous application of PGPR could be used to alleviate the toxicity caused by heavy metals (Tangahu et al., 2011). PGPR have phosphate solubilization ability and release various chelating agents thus affect the mobility and availability of various metals in the rhizosphere and enhance the process of phytoremediation and nutrient transformation. Beside this, these PGPR are also involved in plant growth, maintenance of soil structure, detoxification of hazardous chemicals, and control of plant diseases (He et al., 2007; Oves et al., 2017). In addition, plant microbe interactions provide specific C source to bacteria enabling them to reduce the phytotoxicity in contaminated sites (Khan and Bano, 2016).

Heavy metals toxicity in soils have been increased all over the world which alternately reduced plant growth and productivity. Removal of heavy metals from soil can be done through various plant species (Chibuike and Obiora, 2014). PGPR inoculation efficiently alleviated the heavy metal-induced toxic effect on plant growth and development (Nadeem et al., 2014). Interestingly, significant increase in Cd and Ni contents in shoot of plants treated with 2-PGPRs and SA were (T6) measured. It is documented that SA induced tolerance to various abiotic stresses including heavy metal toxicity (Pál et al., 2002; Rivas-San Vicente and Plasencia, 2011; Oves et al., 2013b). Under stressed condition, SA contents tend to increase in roots of barley plants and induced resistance for Cd (Metwally et al., 2003) The PGPR *P. chinense* P1 was most effective in Ni accumulation, while *B. cereus* strain P2 augmented the accumulation of Cd. However, inoculation mitigated the Ni and Cd toxicity more than Pb. Both, *P. chinense* strain P1 and *B. cereus* strain P2 also had higher Ni and Cd accumulation in the rhizosphere soil compared to T8 and T9. The SA further assisted the PGPR inoculated plants to increase Ni, Pb, and Cd accumulation in the rhizosphere. Similar kind of results were observed by Sayyed et al. (2013) who reported that PGPR accelerated phytoremediation by enhancing the availability of heavy metals to the roots. Findings of Cetin et al. (2011), Tak et al. (2013), and Mishra et al. (2017) are also in accordance with our results.

It is inferred from the results obtained that the 2-PGPR *P. chinense* strain P1 and *B. cereus* strain P2 had higher Cu over Fe also have higher Co and Zn in soil and plant shoot, the effect of SA was to decrease Fe over Cu keeping the Co higher. Cu is an important metal for normal growth and development in plants. It controls many physiological processes, regulates homeostasis, and acts as cofactor for metalloprotiens (Yruela, 2005). It is also involved in the synthesis of various pigments and membrane integrity but it has strong binding ability with organic and inorganic colloids limiting its mobility in soil (Fernandes and Henriques, 1991). Zn and Co are important micronutrients that paly key role in prevention of stunt growth, chlorosis, and spikelet sterility (Singh et al., 2010; Hafeez et al., 2013). Combined application of PGPR and SA increased the accumulation of micronutrients (Cu, Co, and Zn) in both the rhizosphere and shoot of plant. Similar results were recorded by Khan et al. (2017) who demonstrated that SA assists in translocation of micronutrients. Rana et al. (2012) investigated the ability of three rhizosphere bacteria for enhancing the micronutrient content in wheat plant and found significant increase in relation to control. *P. chinense* strain P1 and *B. cereus* strain P2 were shown to increase micronutrient accumulation skills of non-hyper accumulating plants by increasing biomass and growth under stress condition (Armada et al., 2015; Khan and Bano, 2016; Gupta and Kumar, 2017).

Increase in the chlorophyll and carotenoids contents in the leaves of PGPR- and SA-treated plants could be attributed to higher availability of nutrients and increased organic matter in rhizosphere (Esitken et al., 2006; Nadeem et al., 2007). Various reports depicted that PGPR inoculation accelerates the photosynthetic pigments in plants under stress condition (Kohler et al., 2009; Bhattacharyya and Jha, 2012; Heidari and Golpayegani, 2012). Beneficial effects of foliar application of SA on plant growth, chlorophyll content, and accumulation of mineral nutrients under saline condition have also been reported by Yildirim et al. (2008). Rao et al. (1997) and Shirasu et al. (1997) reported the contributory role of SA toward light acclimation and redox homeostasis and found that SA signaling pathway contributes to achieve maximum photosynthetic activity by regulating light acclimation process and redox homeostasis. Carotenoids play a key role in protecting chlorophyll from photo-destruction and facilitate the inoffensive dissipation of excitation energy to light collecting chlorophyll antenna (Young, 1991; Demmig-Adams et al., 1996).

The suppressive effects of PGPR and SA alone or in combination on proline content was noteworthy in the present study. This probably determines the alleviation ability of the osmotic stress and maintenance of bioenergetics of cell under drought stress condition. Further decrease in proline content was obvious in combined application of PGPR and SA (T6) and almost parallel to control. So, PGPR/SA induced drought tolerance and thus no extra proline production was required. PGPR-induced decrease in proline content in leaves of maize and chickpea grown under stressed have been reported by Hameeda et al. (2008) and Khan et al. (2017). Likewise, SA-induced reduction in proline content had also been reported previously (Sakhabutdinova et al., 2003; Krantev et al., 2008). Antioxidant enzymes and MDA content were significantly enhanced under stress condition but follow-up treatment with PGPR/SA considerably lowered the activities of antioxidants and MDA content. The suppressive effect was more dominant in the consortium of PGPR and more so in combined treatment of PGPR and SA. Enhanced activities of antioxidant enzymes and MDA content may be attributed to the fact that PGPR suppress production of reactive oxygen species; consequently lower MDA content or antioxidants. Literature shows the PGPR-induced reductions in the activities of antioxidant enzymes and MDA content in many crops under different stressed conditions (Han and Lee, 2005; Jha and Subramanian, 2013; Singh et al., 2015) In this way, SA proved to be very efficacious in reducing the oxidative damage caused due to MDA content (Verma and Dubey, 1980; Tang et al., 2005; El-Halfawy and Valvano, 2014).

Plant growth promoting rhizobacteria secrete phytohormone in soil that modulates the endogenous levels of phytohormone like IAA and GA (Patten and Glick, 2002; Achard et al., 2009). These hormones are responsible for maintenance of cell division and cell elongation, thus enhance shoot length and root biomass. In this study, significant increases in IAA and GA contents in response to inoculation with PGPR or PGPR/SA consortium were recorded. IAA, which is the main auxin in plants is involved in the modulation of plant growth, embryogenesis, gametogenesis, seedling growth, cell division, flower development, and stimulate shoot elongation and root branching (Zhao, 2010). GA is also involved in the process of plant growth and development, stimulate seed germination, initiation of flowering, overcome dormancy, involved in root formation, and trigger transition from juvenile to adult stage (Mutasa-Göttgens and Hedden, 2009; Gupta and Chakrabarty, 2013). Our results are in line with Fahad et al. (2015) who reported significant increase in phytohormones in wheat plants inoculated with *Bacillus* species. The isolated PGPR appeared stimulatory to shoot and root length and root fresh and dry weights. Application of SA was stimulatory to root and shoot growth and further strengthen the efficacious effect of PGPR on root and shoot growth. Increased root fresh biomass by follow-up application of PGPR and SA could have been associated with increased root length and weight, thereby indicating that the inoculation with PGPR could result in the formation of a much better root system, which favorably affects shoot growth. Similar findings were investigated by Khalid et al. (2004) and Yang et al. (2009), who reported significant increases in plant root and shoot weight and leaf area in different crops inoculated with PGPR. PGPR-induced increase in shoot and root length and root fresh and dry weights had been reported in many plants (Asghar et al., 2002; Ashrafuzzaman et al., 2009; Rivas-San Vicente and Plasencia, 2011; Akhtar et al., 2018). Integrative use of PGPR with SA found to be an effective strategy for the improvement of phytoremediation and plant growth under stress condition.

The PGPR consortia (T5) used alone or in combination with SA significantly enhanced the heavy metal accumulation compared to stress control (T9). However, the consortium of two PGPR (T5) was more effective (40%) for enhancing the accumulation of heavy metals in rhizosphere as compared to foliar application of SA alone (T7). Noteworthy, the combined treatment of two PGPR was more stimulatory for physiological parameters as compared to SA treatment made alone. But the foliar application of SA was more effective for enhancing the activity of antioxidant enzymes and proline content in the leaves of sunflower. SA in combination with PGPR act synergistically for the removal of heavy metals and plant growth and significantly enhanced the remediation abilities and growth of plant as compared to separate treatments of SA and PGPR. T6 (combined treatment of PGPR and SA) was more effective in remediating a higher % of heavy metals as compared to T5 (consortium of two PGPR) and T7 (SA alone). PGPR/SA demonstrated multiple benefits on plant growth and yield because of their role in integrated nutrient management and root proliferation plus exopolysaccharide production (Khan et al., 2018; Naseem et al., 2018). A significant correlation was noted between the Cu, Co, Fe, Zn, Cd, Pb, and Ni of soil and shoot for T5 and T6 treatments, indicating the significance of using SA in combination with bacterial consortia. The accumulation of Cu, Co, Fe, Zn, and Cd was positively and significantly correlated with that of Cu, Co, Fe, Zn, and Cd (*r* = 0.8635, *r* = 9238, *r* = 8146, *r* = 9844, and *r* = 9294) accumulation in shoot. Chlorophyll content was positively and significantly correlated with carotenoid (*r* = 0.8203), protein (*r* = 9091), sugar (*r* = 9236), IAA (*r* = 8334), and GA (*r* = 3631) content but was negatively correlated with proline (*r* = −0.9517), CAT (*r* = −0.9504), APOX (*r* = −0.9707), and SOD (*r* = −0.9045). Carotenoids were highly significantly correlated with IAA (*r* = 0.8973), ABA (*r* = 0.9689), and GA (*r* = 0.9368). Phytohormones IAA, ABA, and GA were positively correlated with chlorophyll (*r* = 0.8334), carotenoids (*r* = 0.9689) and with Cu (*r* = 0.9567), Co (*r* = 0.9611), Fe (*r* = 0.8035), Zn (*r* = 0.9906), Cd (*r* = 0.9780), and Pb (*r* = 0.9202) accumulation in both the rhizosphere and plant shoot. Ghazijahani et al. (2014) reported that foliar application of SA and citric acid changes the root pattern and acquisition of nutrients in the rhizosphere and thus enhances their uptake to plant shoot. Similarly, PGPR modify the root morphology, resulting in greater root surface area for the uptake of nutrients within the soil, and also protect crops against disease (Saravanakumar et al., 2008). These PGPR also produce exopolysaccharides which adhere to soil particles and also act as best matrix for retention of soil moisture, thereby protecting the roots from desiccation (Khan et al., 2017). This could be an additional benefit of PGPR/SA consortium or PGPR, that is, T6 over T5. Consortia of PGPR/SA help in maintaining chlorophyll and relative water content, thus benefiting plants grown under stress condition. It may be due to interaction between plant and beneficial microbes, which improved the nutrient uptake, increase resistance against soil-borne pathogens, and reduce the effect of heavy metals (Gosling et al., 2006).

## Conclusion

The PGPR exhibited increase in the phytoremediation of heavy metals and in the accumulation of other micronutrients. The coinoculation of two PGPR along with SA was very effective for growth parameters as well as for the phytoremediation of heavy metals. The SA in combination with PGPR was effective in enhancing tolerance of plants to drought and heavy metals. The PGPR inoculation showed inhibitory effects on proline, lipid peroxidation, and antioxidant enzymes activity. It is further inferred that the PGPR could be used to enhance the translocation and accumulation of micronutrients and heavy metals in rhizosphere and shoot of plant. The PGPR treatment was also stimulatory for the production of IAA and GA, which assist plants to tolerate stresses.

## Author Contributions

NK and JY designed this project. NK carried out greenhouse and lab experiments and wrote the manuscript.NKand SA performed data analysis. SA and AM improved the grammar and corrected spelling mistakes. PZ, JY, and MA edited the manuscript.

## Conflict of Interest Statement

The authors declare that the research was conducted in the absence of any commercial or financial relationships that could be construed as a potential conflict of interest.
