# Corrigendum: Impact of Salicylic Acid and PGPR on the Drought Tolerance and Phytoremediation Potential of *Helianthus annus*

**DOI:** 10.3389/fmicb.2019.02222

**Published:** 2019-09-27

**Authors:** Naeem Khan, Peiman Zandi, Shahid Ali, Asif Mehmood, Muhammad Adnan Shahid, Jianjun Yang

**Affiliations:** ^1^Department of Plant Sciences, Quaid-i-Azam University, Islamabad, Pakistan; ^2^Institute of Environment and Sustainable Development in Agriculture, Chinese Academy of Agricultural Sciences, Beijing, China; ^3^Plant Epigenetics and Development Lab, Northeast Forestry University, Harbin, China; ^4^Department of Botany, Abdul Wali Khan University Mardan, Mardan, Pakistan; ^5^Horticultural Sciences Department, Institute of Food and Agricultural Sciences, University of Florida, Gainesville, FL, United States

**Keywords:** PGPR, 16S RNA gene sequencing, salicylic acid, phytoremediation, heavy metals

In the original article, we neglected to include funding from the Agricultural Science and Technology Innovation Program of Chinese Academy of Agricultural Science (2016–2020).

In addition, “Jianjun Yang” was not included as an author in the published article. The updated Author Contributions statement appears below:

“NK and JY designed this project. NK carried out greenhouse and lab experiments and wrote the manuscript. NK and SA performed data analysis. SA and AM improved the grammar and corrected spelling mistakes. PZ, JY, and MA edited the manuscript.”

The authors apologize for this error and state that this does not change the scientific conclusions of the article in any way. The original article has been updated.

